# Cadmium isotope fractionation reveals genetic variation in Cd uptake and translocation by *Theobroma cacao* and role of natural resistance-associated macrophage protein 5 and heavy metal ATPase-family transporters

**DOI:** 10.1038/s41438-020-0292-6

**Published:** 2020-05-01

**Authors:** Rebekah E. T. Moore, Ihsan Ullah, Vinicius H. de Oliveira, Samantha J. Hammond, Stanislav Strekopytov, Mark Tibbett, Jim M. Dunwell, Mark Rehkämper

**Affiliations:** 1 Department of Earth Science and Engineering, Imperial College, London, SW7 2BP UK; 2 School of Agriculture, Policy and Development, University of Reading, Reading, RG6 6BZ UK; 3 School of Environment, Earth and Ecosystem Sciences, The Open University, Milton Keynes, MK7 6AA UK; 4 Imaging and Analysis Centre, The Natural History Museum, London, SW7 5BD UK; 5 Present Address: Department of Plant Biology, Institute of Biology, University of Campinas, Campinas, Sao Paulo 13083-970 Brazil; 6 Present Address: National Measurement Laboratory, LGC, Queen’s Road, Teddington, TW11 0LY UK

**Keywords:** Metals, Transgenic plants

## Abstract

In response to new European Union regulations, studies are underway to mitigate accumulation of toxic cadmium (Cd) in cacao (*Theobroma cacao*, Tc). This study advances such research with Cd isotope analyses of 19 genetically diverse cacao clones and yeast transformed to express cacao natural resistance-associated macrophage protein (NRAMP5) and heavy metal ATPases (HMAs). The plants were enriched in light Cd isotopes relative to the hydroponic solution with Δ^114/110^Cd_tot-sol_ = −0.22 ± 0.08‰. Leaves show a systematic enrichment of isotopically heavy Cd relative to total plants, in accord with closed-system isotope fractionation of Δ^114/110^Cd_seq-mob_ = −0.13‰, by sequestering isotopically light Cd in roots/stems and mobilisation of remaining Cd to leaves. The findings demonstrate that (i) transfer of Cd between roots and leaves is primarily unidirectional; (ii) different clones utilise similar pathways for Cd sequestration, which differ from those of other studied plants; (iii) clones differ in their efficiency of Cd sequestration. Transgenic yeast that expresses *TcNRAMP5* (*T. cacao* natural resistance-associated macrophage gene) had isotopically lighter Cd than did cacao. This suggests that NRAMP5 transporters constitute an important pathway for uptake of Cd by cacao. Cd isotope signatures of transgenic yeast expressing HMA-family proteins suggest that they may contribute to Cd sequestration. The data are the first to record isotope fractionation induced by transporter proteins in vivo.

## Introduction

Cadmium (Cd) is highly toxic to humans as it accumulates in the body to cause chronic damage to kidneys and bones, and it is classified as a Group 1 carcinogen by the International Agency for Research on Cancer, with no safe level of exposure^1^. Many efforts are underway to restrict exposure of humans to this metal by limiting the amount present in drinking water^2^ and in the diet^3^. Crops that represent significant dietary sources of Cd include rice^2^, leafy^4–6^ and other vegetables^7^, and also cacao (*Theobroma cacao*), the beans of which are the source of chocolate. The Cd contents of cacao beans show large regional variations, with the highest levels reported for material from South and Central America^8–13^. In this region, the Cd concentrations of cacao beans commonly exceed 0.6 to 0.8 mg kg^−1 ^^10^, which is the approximate upper range for cocoa products that meet the new European Union (EU) regulations that came into force in 2019^14^. As such, there is an urgent imperative to address this issue.

Short-term approaches designed to reduce Cd uptake in cacao include agronomic changes, such as the application of biochar and/or lime to lower the bioavailability of Cd^15^, while differences in Cd accumulation between cacao genotypes have also been identified^16,17^. In the longer term, genetic approaches may hence provide a means to reduce the Cd contents of cacao and other crops, and numerous recent studies have investigated this potential, including for cacao, rice, wheat and tobacco.

Such work has particularly focused on genes that encode either transporters, such as natural resistance-associated macrophage proteins (NRAMPs)^18–25^, which mediate the entry of Cd from the rhizosphere, or those such as heavy metal ATPases (HMAs)^26–32^, which determine the deposition of Cd in vacuoles or its translocation in the plant. Significant findings include (i) that the level of NRAMP5 in rice may be linked to the higher uptake of Cd in this species than in maize^21^, (ii) combining knockout of one homeologous *HMA4* gene (nonsense mutation) and reduction of the other (missense mutation) reduced Cd levels in tobacco, while maintaining plant vigour^27^, and (iii) the identification in wheat of *HMA3* variants linked to reduced Cd accumulation^26^. This latter example led to the development of a kompetitive allele-specific polymerase chain reaction marker that is now used in a marker-assisted breeding programme^33^.

Most previous investigations of Cd accumulation in crops relied solely on measurements of Cd concentration. More recently, however, coupled Cd stable isotope and concentration measurements have been used to trace the origin of isotopically distinct Cd inputs into soils and to gain novel insights into the cellular and molecular processes that are employed by plants for uptake, internal transport and sequestration of Cd. The first investigations thereby revealed that wheat and barley display similar trends of Cd isotope fractionation^34,35^, which differ significantly from those observed for Cd-tolerant and hyperaccumulator plants^36^. A recent pilot field study conducted in Ecuador was the first investigation to employ Cd isotope analyses to examine soil-cacao systems, and this provided a number of promising results^37^. In detail, the isotope compositions of topsoil Cd enrichments suggested that these were most likely derived from decaying tree litter that was employed as an organic fertiliser. In addition, the analyses revealed differences in the Cd isotope fingerprints of the cacao leaves and beans that were tentatively linked to the different cacao cultivars that were analysed.

The current study builds on this pilot work, employing coupled Cd isotope and concentration measurements to further investigate possible genetic differences in the uptake and translocation of Cd by different cacao clones and to better constrain the molecular pathways that are responsible for the enrichment of Cd in cacao plants. To this end, the investigation encompasses analyses of roots and leaves from 19 genetically diverse cacao clones that were grown under controlled hydroponic conditions. Additional analyses were carried out for transgenic yeast cells that were modified to express cacao genes, which encode the NRAMP5- and HMA-family transporter proteins. As such, this study represents the first direct in vivo investigation of the trace metal stable isotope fractionation that is induced by specific transporter proteins.

## Results

### Quality control

Total procedural blanks were between 30 and 170 pg Cd during the course of the study. As this corresponds to <0.1% of indigenous Cd in the samples, no corrections were applied to the measured Cd isotope compositions. The Cd isotope data of samples were assigned error bars based on the analytical precision determined for repeat analyses of the SRM 3108 Cd isotope standard, which bracketed the sample runs. This typically yielded 2 s.d. (standard deviation) precisions between ±0.04‰ and ±0.08‰ (Table 1).

**Table 1:** Cd contents, concentrations and isotope compositions of leaves, roots and calculated total plants for 19 cacao genotypes grown in hydroponic solution with 20 µmol L^−1^ CdCl_2_ (and δ^114/110^Cd = −0.36 ± 0.04)

Cacao clones	Leaves				Roots				Total plant			Isotope fractionation	
	*f*	[Cd] (µg g^−1^)	δ^114/110^Cd (‰)	2 s.d. (‰)	*f*	[Cd] (µg g^−1^)	δ^114/110^Cd (‰)	2 s.d. (‰)	Cd (μg)	[Cd] (µg g^−1^)	δ^114/110^Cd (‰)	Δ^114/110^${\rm{Cd}}_{{\rm{t}}-{\rm{s}}^{\rm{b}}}$ (‰)	Δ^114/110^${\rm{Cd}}_{1-{\rm{t}}^{\rm{c}}}$ (‰)
B 5/7 [POU]	0.40	142	−0.46	0.06	0.22	475	−0.82	0.03	198	222	−0.61	−0.25	0.15
Catie 1000	0.47	361	−0.48	0.06	0.30	551	−0.73	0.05	235	426	−0.58	−0.22	0.10
CC 41 (1)	0.65	266	−0.58	0.06	0.09	163	−0.73	0.05	189	238	−0.61	−0.25	0.03
CC 41 (2)	0.74	291	−0.65	0.06	0.09	195	−0.71	0.05	271	270	−0.61	−0.25	−0.04
CL 19/10	0.49	112	−0.51	0.07	0.24	478	−0.75	0.02	313	173	−0.60	−0.24	0.09
GU 207/H	0.53	182	−0.53	0.06	0.32	300	−0.72	0.06	86.3	217	−0.61	−0.25	0.08
GU 263/V	0.57	403	−0.64	0.10	0.27	433	−0.82	0.07	233	413	−0.70	−0.34	0.06
IMC 27	0.27	139	−0.35	0.09	0.43	501	−0.72	0.04	155	267	−0.57	−0.21	0.21
LP 1/41 [POU]	0.36	171	−0.50	0.04	0.25	757	−0.68	0.04	305	306	−0.58	−0.22	0.08
Matina 1–7^a^	0.04	3.38	−0.20	0.08	0.49	207	−0.68	0.09	113	52	−0.55	−0.19	0.35
NA702	0.38	78.2	−0.51	0.07	0.30	368	−0.64	0.07	241	140	−0.57	−0.21	0.06
PNG 340	0.20	29.7	−0.13	0.07	0.32	220	−0.53	0.07	142	83.8	−0.35	0.01	0.22
POUND 12/A [POU]	0.60	338	−0.46	0.05	0.20	497	−0.74	0.13	296	376	−0.54	−0.18	0.08
RB 46	0.36	199	−0.50	0.04	0.39	825	−0.68	0.04	284	361	−0.59	−0.23	0.09
RIM 189 [MEX]	0.19	30.4	0.02	0.10	0.50	348	−0.68	0.06	207	107	−0.44	−0.08	0.46
SCA 9	0.31	68	−0.40	0.05	0.31	375	−0.62	0.03	150	142	−0.51	−0.15	0.11
SPA 9 [COL]	0.62	300	−0.57	0.05	0.14	275	−0.76	0.04	258	293	−0.62	−0.26	0.05
TARS 31	0.44	355	−0.44	0.04	0.37	565	−0.71	0.04	431	433	−0.57	−0.21	0.13
TSA 654	0.57	298	−0.67	0.05	0.23	541	−0.76	0.04	323	356	−0.70	−0.34	0.03
U 70 [PER]	0.61	493	−0.60	0.05	0.18	519	−0.85	0.05	200	500	−0.67	−0.31	0.07
Min/max	0.04/0.74	3.4/493	−0.67/0.02		0.09/0.50	163/825	−0.85/−0.53		86/431	52/500	−0.70/−0.35	−0.34/0.01	−0.04/0.46
Mean	0.44	213	−0.46		0.28	430	−0.72		231	269	−0.58	−0.22	0.12
s.d.	0.17	136	0.17		0.11	174	0.07		80	125	0.08	0.08	0.11

Also shown are the isotope fractionations between total plants and growth solution (Δ^114/110^Cd_tot-sol_) as well as leaves and total plants (Δ^114/110^Cd_leaf-tot_). CC 41 (1) and (2) are two separate plants of the same clone.

*f* mass fraction of Cd in leaves or roots relative to the Cd inventory of the total plant, *s.d.* standard deviation.

^a^Mean data from analyses of two separate samples from the same Matina 1–7 plant (see Supplementary Table S1 for individual results), ^b^tot-sol, ^c^leaf-tot

The Cd isotope and concentration data that were obtained for relevant reference and quality control materials are in excellent agreement with certified values and/or published results (see Supplementary Table S1). Replicate analyses of BAM I012 Cd (*n* = 16) and NIST Spinach Leaf SRM 1570a (*n* = 4) provide δ^114/110^Cd data with a reproducibility of ±0.06‰ (2 s.d.; the reproducibility of all isotopic data is quoted in the same manner in the following). Similarly, replicate analyses of the Sigma-Aldrich CdCl_2_, which was added to the hydroponic solutions and the yeast culture medium, yielded δ^114/110^Cd = −0.36 ± 0.04‰. The somewhat poorer reproducibility obtained for the in-house cocoa leaf quality control material (±0.13‰) most likely reflects minor sample heterogeneity, which is also apparent in the Cd concentration results for this sample (Supplementary Table S1).

To assess sample homogeneity and biological variability for the cacao clone samples, analyses were carried out on duplicate root and leaf aliquots from a single plant of Matina 1–7 and two separate plants of the CC 41 clone. Notably, the concentration data show reasonable agreement, which demonstrates that the results for two samples of a single plant or two different plants of the same clone are substantially less variable than the overall range of Cd concentrations that were determined for the leaves and roots of different clones. The δ^114/110^Cd values of the leaf and root replicates for both genotypes are, furthermore, identical within the analytical precision (Table 1 and Supplementary Table S1). These observations are in accord with the results of previous work, which shows that (i) plant experiments conducted in hydroponic systems under controlled conditions generally yield highly reproducible results for metal uptake studies^33^ and (ii) Cd isotope analyses of biological replicates nearly consistently achieve a reproducibility similar to or only slightly worse than the analytical precision of the measurements^34–37^. These observations, as well as the high cost and effort of the Cd isotope measurements, justify that no further replicate analyses were carried for samples in this study.

### Cadmium data for cacao plants

Details of plant weights can be found in Supplementary Table S2. In summary, seedlings grew to a total dry mass (root, stem and leaves) between 0.4 and 2.2 g, whereby roots and leaves constituted 9–28% and 52–75% of the total mass, respectively. In the following, the Cd concentration and isotope data are summarised alongside the tissue Cd contents. To enable full mass balance calculation for the plants, the stems were assigned Cd concentrations and δ^114/110^Cd values at the midpoint of the results obtained for roots and leaves, in accord with literature data (see Supplementary Information Note 1 for details).

**Fig. 1:**
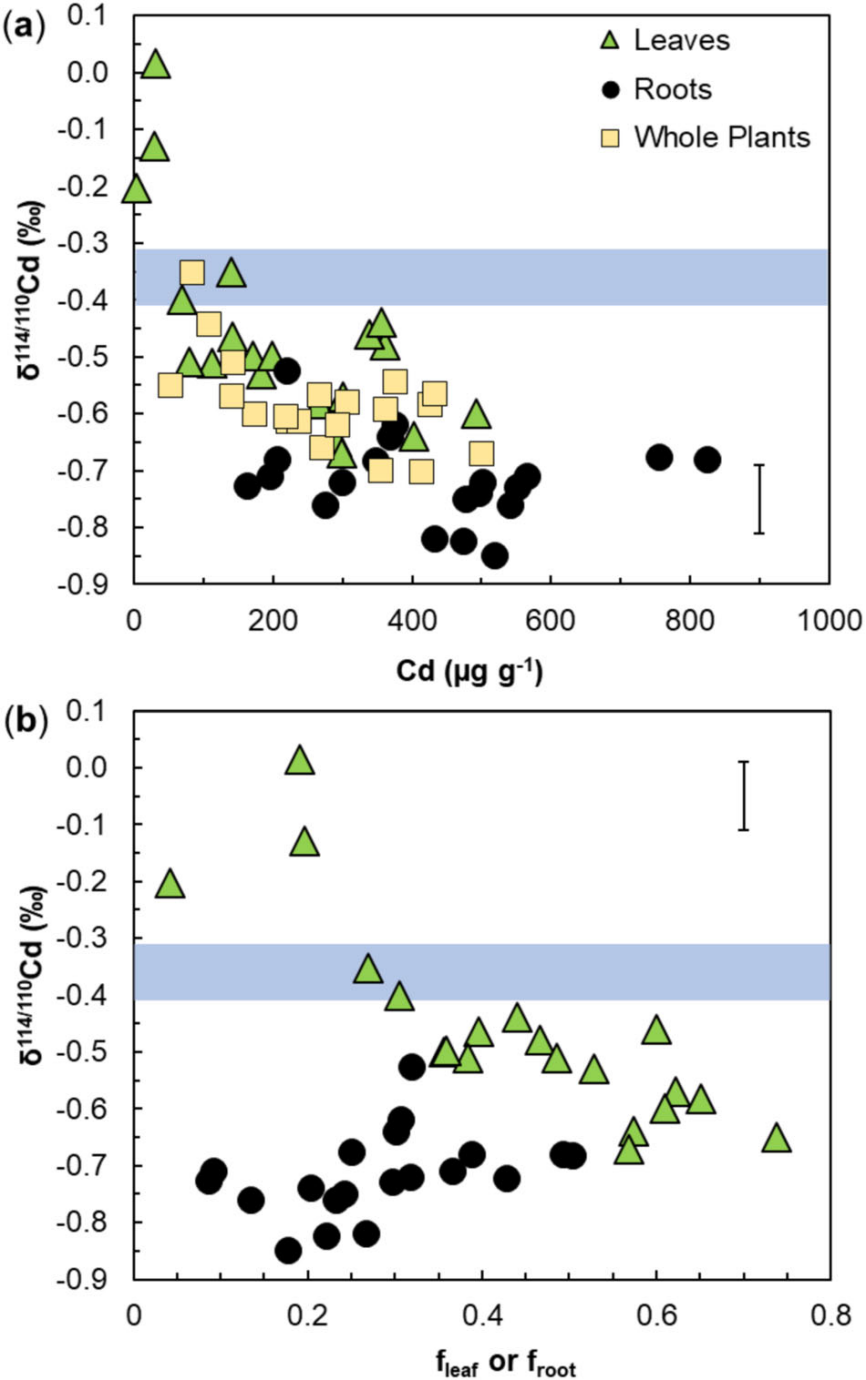
Cadmium concentrations, mass fractions and stable isotope compositions of hydroponically-grown cacao plants. Plots show Cd isotope compositions (as δ^114/110^Cd) for leaves, roots (and whole plants, where applicable) versus **a** Cd concentrations and **b** mass fractions (*f*) of Cd in leaves (*f*_leaf_) and roots (*f*_root_) relative to the Cd inventory of the whole plants. The blue bars denote the Cd isotope composition of the hydroponic solutions that contained 20µmolL^−1^ CdCl_2_. A typical ±0.06‰ error bar is shown for the δ^114/110^Cd data

The cacao leaves had Cd contents Cd_leaf_ and mass fractions *f*_leaf_ (relative to the total plant Cd inventory) that exceeded Cd_root_ and *f*_root_ for 14 of the 19 genotypes, with mean values of *f*_leaf_ = 44% and *f*_root_ = 28% (Table 1). In contrast, the Cd concentrations of leaves [Cd]_leaf_ are lower than [Cd]_root_ for 16 genotypes. With results in the range 3.4–493 µg g^−1^ for [Cd]_leaf_ and 163–825 µg g^−1^ for [Cd]_root_, the latter are notably less variable (Fig. 1a, Table 1).

The leaves of all clones had higher δ^114/110^Cd_leaf_ compared to δ^114/110^Cd_root_, whereby the leaf data are about twice as variable compared to the latter, both in terms of overall range and s.d. (Table 1). The δ^114/110^Cd_root_ values are furthermore consistently lower than δ^114/110^Cd_sol_ of the hydroponic growth solution (Fig. 1a, b). Notably, the leaf data show scattered but clear correlations of increasing δ^114/110^Cd_leaf_ with decreasing values for [Cd]_leaf_, *f*_leaf_ (Fig. 1) and Cd_leaf_ (Supplementary Fig. S1). While no such correlations are apparent for the root results, the δ^114/110^Cd_tot_ values also appear to show a small but systematic increase with decreasing [Cd]_tot_ (Fig. 1a).

### Cadmium data for expression of cacao genes in yeast

**Fig. 2:**
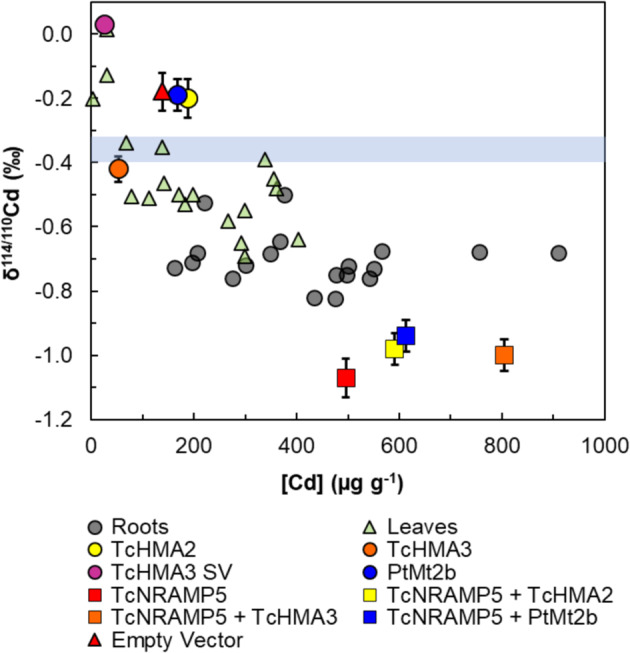
Cadmium isotope compositions and concentrations for transgenic yeasts. Results are shown for yeasts transformed to express either one or two genes from cacao (Tc) or poplar (Pt), which encode expression of metal transporters or sequestering proteins from the NRAMP, HMA and Mt families, in comparison to the control (yeast with the empty vector). Also shown are the data for leaves and roots of 19 cacao clones. The blue bar represents the Cd isotope composition of the CdCl_2_ that was added to the yeast culture medium and the hydroponic solution of the cacao plants. Error bars represent the 2s.d. precision of replicate standard measurements that bracketed the sample analyses

The yeast grew to (dry) weights between 4.4 and 23 mg, where the yeast modified with the empty vector (EV); *PtMt2b* (*Populus* metallothionein 2b), *TcHMA2* (*T. cacao* heavy metal ATPase 2) and *TcHMA3 SV* (a splice variant of *TcHMA3*) had substantially higher final masses than the other samples (Table 2). Both the Cd concentrations and isotope compositions of the yeast showed substantial variability, with results ranging from 26 to 805 µg g^−1^ for [Cd] and from −1.07‰ to 0.03‰ for δ^114/110^Cd. The results thereby fall into two groups (Fig. 2). The yeast modified with EV shows a minor enrichment of heavy Cd isotopes (of 0.18‰) relative to the culture medium, and the single *TcHMA2*, *TcHMA3* and *PtMt2b* transformants resemble the EV yeast to within ±0.25‰ or less. In addition, these samples also have relatively low Cd concentrations of under 200 µg g^−1^. In contrast, yeast transformed with *TcNRAMP5* (*T. cacao* natural resistance-associated macrophage gene), either alone or together with *TcHMA2*, *TcHM3* or *PtMt2b*, have δ^114/110^Cd values that are lower than those of the culture medium and EV by about 0.60‰ and 0.75‰, respectively, and higher Cd concentrations of ~500 to 800 µg g^−1^ (Fig. 2, Table 2).

**Table 2:** Weights, Cd contents and isotope concentrations for yeast cells grown in culture media with 2 µmol L^−1^ CdCl_2_ (and δ^114/110^Cd = 0.36 ± 0.04‰)

Construct	Dry weight (mg)	Cd_tot_ (µg)	[Cd] (µg g^−1^)	δ^114/110^Cd (‰)	2 s.d. (‰)	Δ^114/110^Cd_trans-ev_ (‰)
Empty vector	19	2.62	138	−0.18	0.09	0
TcHMA2	23	4.32	188	−0.20	0.06	−0.02
TcHMA3	4.4	0.231	52.5	−0.42	0.04	−0.24
TcHMA3 SV	21	0.537	25.6	0.03	0.02	0.21
PtMt2b	23	3.86	168	−0.19	0.05	−0.01
TcNRAMP5	5.6	2.78	496	−1.07	0.06	−0.89
TcNRAMP5 + TcHMA2	9.7	5.73	591	−0.98	0.05	−0.80
TcNRAMP5 + TcHMA3	6.9	5.55	805	−1.00	0.05	−0.82
TcNRAMP5 + PtMt2b	9.7	5.94	613	−0.94	0.05	−0.76

Also shown is the apparent isotope fractionation Δ^114/110^Cd_trans-ev_ between transgenic yeast modified to express cacao proteins and the control (transformation with the empty vector).

*s.d.* standard deviation

**Fig. 3:**
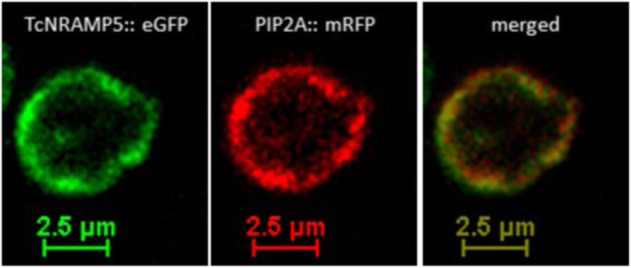
TcNRAMP5-eGFP localisation in yeast cells. Confocal microscopy images of the yeast cells expressing TcNRAMP5-eGFP and PIP2A-mRFP are shown. Left: TcNRAMP5-eGFP fluorescence in green. Centre: PIP2A-mRFP fluorescence in red. Right: merged image, in which the yellow colour results from the overlap of red and green

Fluorescence images obtained by confocal microscopy demonstrate that the cacao TcRAMP5 protein expressed in transgenic yeast cells is localised in the external plasma membrane, as expected (Fig. 3).

## Discussion

### Isotope fractionation associated with Cd uptake by cacao

Evaluation of the Cd isotope data reveals that the cacao clones (total plants) are consistently (with one exception) isotopically lighter than the hydroponic solutions in which they were cultured (Fig. 1a). As such, the clones show a mean Cd isotope fractionation between total plants and solution of Δ^114/110^Cd_tot-sol_ = −0.22 ± 0.08‰ (Table 1). Similar isotope systematics were previously observed in comparable Cd uptake experiments with Cd-tolerant *Ricinus communis* and Cd hyperaccumulator *Solanum nigrum*^36^ (for simplicity, these cultivars are simply termed as ‘Cd accumulators’ in the following discussion). In detail, replicate experiments with the Cd accumulators also yielded lighter Cd isotope compositions for the whole plants in comparison to the hydroponic growth solutions, but the observed isotope fractionation was more pronounced with Δ^114/110^Cd_tot-sol_ values between −0.27‰ and −0.48‰^36^.

The distinct Cd isotope fingerprints that are recorded by both the cacao clones and the Cd accumulators reflect the isotope fractionation that is imparted by the plants during root uptake of the metal, and, possibly, any subsequent root excretion of Cd. While recent experiments revealed transporters that facilitate Cd efflux from rice roots^38,39^, it is not known whether such efflux transporters are also active in cacao roots. Furthermore, even in an upregulated state, such transporters appeared to reduce the Cd concentrations of the rice roots by <20%. As such, it is reasonable to conclude that the Cd isotope compositions of the cacao clones and the Cd accumulators are primarily imparted during Cd uptake.

The distinct Δ^114/110^Cd_tot-sol_ fingerprints, which are recorded by cacao versus Cd accumulators, may thus reflect the presence of different Cd uptake transporters that impart characteristic Cd isotope signatures. An alternative, and more likely, scenario is that different plant groups utilise several similar transporters for Cd uptake. Each transporter thereby imparts a characteristic isotopic signal during Cd handling, and this could vary from preferred transport of light Cd isotopes to little or even positive Cd isotope fractionation, if the transporters feature different metal coordination environments^34,40^.

In this case, the distinct Δ^114/110^Cd_tot-sol_ signatures of cacao plants and Cd accumulators arise because Cd uptake by the latter is governed to a greater extent by transporters that produce preferential uptake of isotopically light Cd. Given that the Cd accumulators also achieve higher Cd bioconcentration factors than the cacao clones^41^, it is conceivable that metal transporters, which are particularly efficient in facilitating Cd uptake by roots, also generate particularly negative Cd isotope fractionations.

### Isotope fractionation associated with Cd translocation

The cacao leaves ubiquitously have heavier Cd isotope compositions than the roots, whereby the former are considerably more variable, as δ^114/110^Cd increases with decreasing [Cd]_leaf_ and *f*_leaf_ (Fig. 1a, b). As a consequence, the apparent isotope fractionation between leaves and roots, Δ^114/110^Cd_leaf-root_, also increases systematically with decreasing *f*_leaf_ from values of ~0.0‰ to 0.7‰ (Supplementary Fig. S2). The higher δ^114/110^Cd_leaf_ values are thereby most readily explained by retention of isotopically light Cd in the roots (and stems) and preferential translocation of isotopically heavier Cd to the leaves. The results furthermore reveal large differences between the clones in the extent to which Cd is retained in roots rather than translocated to leaves. Efficient root retention of Cd is thereby clearly marked by particularly positive δ^114/110^Cd_leaf_ values (Fig. 1a, b).

It was previously shown for wheat that the translocation of Cd from roots to stems and leaves can be treated, to a first order, as a unidirectional flow-through system. In this case, the isotope fractionation recorded by the mobilised Cd is a consequence of partial Cd sequestration in roots (or other plant organs) prior to or during translocation. The coupled variations of Cd concentrations and isotope compositions that are expected for such a system can be described with the Rayleigh equation for closed-system isotope fractionation:

1 $${\updelta}^{114/110}{\mathrm{Cd}}_{{\mathrm{mob}}} = {\updelta}^{114/110}{\mathrm{Cd}}_{{\mathrm{tot}}} + \Delta ^{114/110}{\mathrm{Cd}}_{{\mathrm{seq}} {\mbox{-}} {\mathrm{mob}}}{\mathrm{ln}}\left( {{f}_{{\mathrm{mob}}}} \right)$$

where δ^114/110^Cd_mob_ and δ^114/110^Cd_tot_ denote the Cd isotope compositions of the mobilised Cd and the total plant, respectively, *f*_mob_ represents the mass fraction of mobilised Cd relative to the total Cd inventory of the plant, and Δ^114/110^Cd_seq-mob_ records the isotope fractionation between the sequestered and mobile Cd. Notably, Eq. 1 can be utilised together with the data of this study to define the fractionation Δ^114/110^Cd_seq-mob_ that is generated in cacao by storage of Cd in roots and stems, while mobile Cd is moved to the leaves. If the Cd mobility is indeed, to a first order, unidirectional from roots to leaves and in accord with closed-system dynamics, then:

2 $$\Delta ^{{\mathrm{114/110}}}{\mathrm{Cd}}_{{\mathrm{leaf}} {\mbox{-}} {\mathrm{tot}}} = \Delta ^{{\mathrm{114/110}}}{\mathrm{Cd}}_{{\mathrm{seq}} {\mbox{-}} {\mathrm{mob}}}{\mathrm{ln}}\left({{f}_{{\mathrm{leaf}}}} \right)$$

**Fig. 4:**
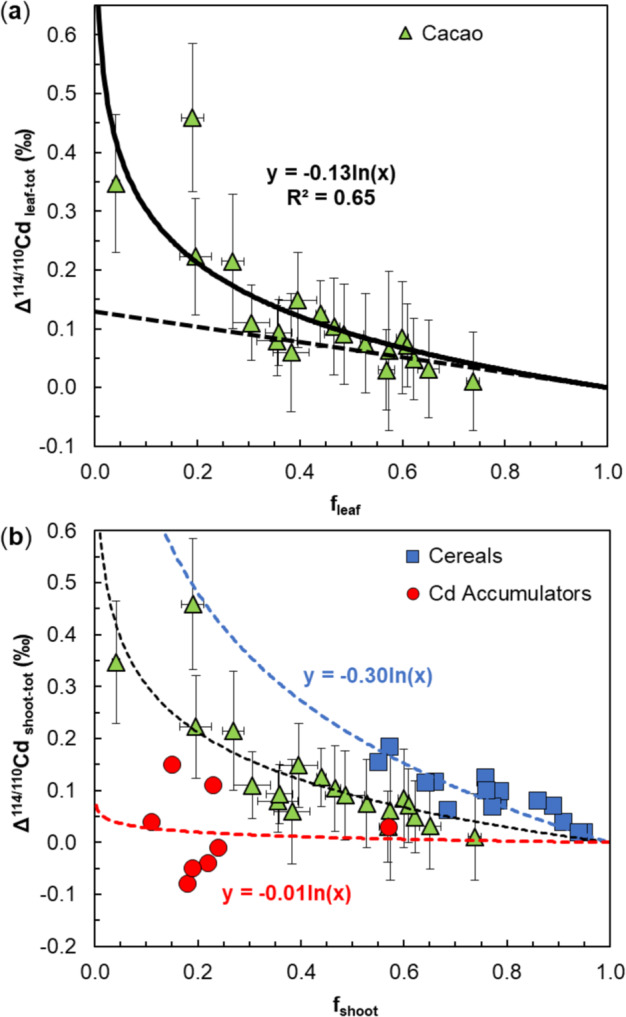
Cadmium isotope fractionation in cacao, cereals and Cd accumulators. **a** Plot of Cd isotope fractionation Δ^114/110^Cd_leaf-tot_ between cacao leaves and total plants versus mass fraction of Cd present in leaves. The solid line denotes a best-fit Rayleigh fractionation trend that is forced through Δ^114/110^Cd_leaf-to:_ = 0 at *f*_leaf_ = 1. This yields an isotope fractionation of Δ^114/110^Cd_seq-mob_ = −0.13‰ between Cd sequestered in roots/stems and Cd mobilised to leaves. The dashed line denotes an open-system isotope fractionation trend with Δ^114/110^Cd_seq-mob_ ≈ −0.13‰; this trend is in accord with some ‘outlier’ results, but provides a poor overall fit for the cacao data. **b** Plot of Cd isotope fractionation Δ^114/110^Cd_shoot-tot_ between shoots and total plants versus the mass fraction of Cd present in shoots based on literature data for cereals (wheat, barley)^33,34^ and Cd accumulators^35^. The blue and red dashed lines denote best-fit Rayleigh fractionation trends for the two datasets, which are forced through Δ^114/110^Cd_shoot-tot_ = 0 at *f*_shoot_ = 1. The trends define Δ^114/110^Cd_seq-mob_ fractionations of −0.30‰ for the cereals and −0.01‰ for the Cd accumulators

Notably, a best-fit line for the cacao clone data in a plot of Δ^114/110^Cd_leaf-tot_ versus *f*_leaf_ (and forced through Δ^114/110^Cd_leaf-tot_ = 0 at *f*_leaf_ = 1) captures nearly all of the results to yield a well-defined Δ^114/110^Cd_seq-mob_ = −0.13‰ (Fig. 4a). Two important issues deserve to be discussed in the context of the finding that most cacao clone data fit well to single mass fractionation trend.

First, the observation suggests that the transfer of Cd between roots and leaves in cacao plants is indeed primarily unidirectional, with no or only limited remobilisation of Cd once it reaches the leaves. This is supported by results from a recent field study of cacao^37^, which found high Cd concentrations for both cacao leaves and leaf litter and very similar Cd isotope compositions. The latter data suggest that Cd continues to accumulate even in older leaves and does not redistribute with onset of senescence.

However, the results for some cacao clones may not be fully in accord with the best-fit Rayleigh isotope fractionation trend (Fig. 4a). This pertains, in particular, to a group of four clones that plot underneath the logarithmic trend at *f*_leaf_ ≈ 0.3–0.4. Notably, these clones are in accord with a linear fractionation trend, which would be expected for open-system isotope fractionation as a consequence of bi-directional movement of Cd between roots and leaves:

3 $$\Delta ^{{\mathrm{114/110}}}{\mathrm{Cd}}_{{\mathrm{leaf}} {\mbox{-}} {\mathrm{tot}}} \, = \, \Delta ^{{\mathrm{114/110}}}{\mathrm{Cd}}_{{\mathrm{seq}} {\mbox{-}} {\mathrm{mob}}}\left( {{1 - f}_{{\mathrm{leaf}}}} \right)$$

Interestingly, the open-system fractionation trend that accounts for the four ‘outlier’ clones (and is forced through Δ^114/110^Cd_leaf-tot_ = 0 at *f*_leaf_ = 1) also yields Δ^114/110^Cd_seq-mob_ ≈ −0.13‰. As the open- and closed-system fractionation lines fall close to one another at *f*_leaf_ > 0.5, most clones are in fact in accord with both inferred fractionation trends. Given the constraints, however, the overall fit of the data is considerably better for the closed-system fractionation model, and this indicates that the Cd partitioning and isotope fractionation within cacao is dominated, at least in most clones, by unidirectional Cd transport and closed-system dynamics.

Second, the excellent fit of nearly all cacao data to fractionation models with Δ^114/110^Cd_seq-mob_ = −0.13‰ suggests that the different cacao clones utilise similar biochemical pathways for the sequestration of Cd in roots and stems. As such, the clones primarily differ in the efficiency of Cd sequestration, most likely due to genetic differences in the extent to which the sequestration mechanism is expressed.

Of further interest is a comparison of the inferred fractionation of Δ^114/110^Cd_seq-mob_ = −0.13‰ for cacao with equivalent results for other plants. Available for such a comparison are data for the Cd partitioning within cereals (wheat, barley) and Cd accumulator plants^34–36^. While a direct comparison of these results is not feasible, given that the current investigation did not encompass analyses of the plant stems and as the cereal studies differentiated the above ground parts only into straw and grains, there are still a number of important observations. In detail, the wheat and barley data yield a well-defined Δ^114/110^Cd_seq-mob_ = −0.30‰ (when forced through Δ^114/110^Cd_shoot-tot_ = 0 at *f*_shoot_ = 1) for the mobilisation Cd into the shoots (grains + straw; Fig. 4b). In contrast, the data for the Cd accumulators show significantly more scatter, but the nearly horizontal fractionation trend with Δ^114/110^Cd_seq-mob_ = −0.01‰ for the mobilisation of Cd into the shoots (stem + leaves) clearly contrasts with the steeper correlations defined by the cacao clones and cereals (Fig. 4b).

Considered together, these results are intriguing as they suggest that the three investigated plant groups (19 cacao clones, the cereals wheat and barley, and the Cd accumulators) are each characterised by a distinct Δ^114/110^Cd_seq-mob_ fractionation signature, as defined by the systematic within-group variation of Δ^114/110^Cd_shoot-tot_ with *f*_leaf_ (Fig. 4b). This implies that plants from each group employ a roughly similar molecular strategy for the internal sequestration of Cd, which is expressed to different extents. In contrast, the distinct Δ^114/110^Cd_seq-mob_ values that are found for the three groups demonstrate that they utilise different characteristic Cd sequestration strategies. The latter may involve use of either different proteins for binding of Cd or several groups of proteins (that may be similar between the plant groups), which generate distinct Cd isotope signatures and that are employed to different extents to facilitate Cd retention.

### Role of metal transporters in response to Cd exposure in cacao

The analyses carried out on the transgenic yeast are particularly significant, as they constitute the first direct in vivo investigation of trace metal stable isotope fractionation induced by specifically targeted transporter proteins.

Yeast cells transformed with *TcNRAMP5* genes, alone or in combination with *TcHMA2*, *TcHMA3* or *PtMt2b*, generated Δ^114/110^Cd_trans-ev_ values between −0.76‰ and −0.89‰ (Table 2, Fig. 2). Notably, the negative fractionation with an enrichment of light Cd isotopes in the product resembles the mean fractionation of Δ^114/110^Cd_tot-sol_ = −0.22 ± 0.08‰ (Table 1) observed for the cacao clones relative to the hydroponic solution. Together with the observations that (i) the yeast with *TcNRAMP5* genes also have relatively high Cd concentrations (Fig. 2) and (ii) the expressed TcNRAMP5 proteins are localised in the external plasma membrane (Fig. 3), there is strong evidence that NRAMP5 transporters play an important role in facilitating uptake of isotopically light Cd by the roots of cacao plants.

The results also suggest, however, that root uptake of Cd by cacao is not solely facilitated by TcNRAMP5 transporters. This conclusion stems from the observation that the yeast modified with *TcNRAMP5* genes consistently feature a stronger preference for uptake of isotopically light Cd (with Δ^114/110^Cd_trans-ev_ ≈ −0.8‰) than the cacao clones, which display Δ^114/110^Cd_tot-sol_ ≈ −0.2‰ (Table 1). It is conceivable that this disparity in the magnitude of isotope fractionation is primarily due to differences in how Cd is handled and stored within unicellular yeast versus the much more complex cacao plant. However, given that the comparison focuses on the total net Cd uptake of both organisms, the difference most likely reflects that Cd uptake by cacao roots is facilitated not only by NRAMP proteins.

If additional Cd uptake pathways feature coordination environments with a lower preference for or even a discrimination against isotopically light Cd, this could readily explain the somewhat variable but still consistent Cd uptake signature of the cacao clones with Δ^114/110^Cd_tot-sol_ = −0.22 ± 0.08‰ (Table 1). As such, NRAMP proteins are probably an important but not the sole membrane transporter responsible for the Cd uptake of cacao. An alternative scenario is that the relatively low Δ^114/110^Cd_tot-sol_ values of the cacao clones also record the activity of Cd efflux transporters, with a preference for excretion of isotopically light Cd, in addition to NRAMP5-facilitated Cd uptake. Additional expression experiments with transgenic organisms, preferably plants, which are modified with various transporter proteins that might be involved in uptake and efflux of Cd by cacao, are thus desirable to further examine the suggested interpretations.

The results for the transgenic yeast are also of interest in the context of the finding that the variable, but consistently positive δ^114/110^Cd_leaf_ values, are a consequence of retention of isotopically light Cd in the roots and (by inference) stems, and mobilisation of isotopically heavy Cd to the leaves. The molecular origin of this sequestration is thereby constrained by the cacao clone data, which demonstrate that this process imparts an isotope fractionation of Δ^114/110^Cd_seq-mob_ = −0.13‰ (Fig. 4a).

Given that the fluorescence imaging in this study confirms that NRAMP5 is localised in cell membranes, it is unlikely that this protein is responsible for Cd sequestration. More likely candidates for this are the HMA-family proteins. This conclusion is supported by the observation that the yeast, which were doubly transformed with *TcNRAMP5* plus either *TcHMA2*, *TcHMA3* or *TcHMA3 SV* genes feature by far the highest Cd concentrations of the dataset, but δ^114/110^Cd values that are analytically identical to the yeast modified only with *TcNRAMP5* (Table 2, Fig. 2). As the singly transformed yeast that encodes *HMA3* and *HMA3 SV* clearly records Cd isotope fractionation relative to the EV control, the results imply that the HMA-family proteins are not involved in the uptake of Cd, unless they can impart the same isotopic signature as *TcNRAMP5* due to essentially identical coordination environments, which is unlikely. As such, the high Cd concentrations of the doubly modified yeast record the enhanced ability of the cells both to take up Cd (via NRAMP transporters) and to sequester the metal via HMA-family proteins once it has entered the cells.

The isotopic data obtained for the yeast singly transformed with three different *HMA*-family genes are of interest because of their moderately variable δ^114/110^Cd values that differ by <0.25‰ from the control (Fig. 2, Table 2). As such, the results indicate that Cd sequestration by HMA proteins is associated with no resolvable isotope fractionation (TcHMA2) or a modest enrichment of Cd that is either isotopically heavy (TcHMA3 SV) or isotopically light (TcHMA3). As such, these results suggest that Cd sequestration in cacao, which imparts a characteristic fractionation of Δ^114/110^Cd_seq-mob_ = −0.13‰ across the investigated clones, can be readily ascribed to the concerted action of the HMA-family proteins that were studied here. Further experiments with transgenic organisms (preferably plants) modified with *HMA*-family genes and efforts to localise HMA proteins in cells are needed, however, to substantiate this tentative conclusion.

Of further significance is the finding that the yeast transformed with *HMA2* and *PtMt2b* genes have identical δ^114/110^Cd values, within error, and record no isotope fractionation relative to the EV control (Fig. 2, Table 2). Metallothioneins are known to be involved in the sequestration of metals within cells^42^, so the transformation of the yeast with *PtMt2b* is not expected to increase the influx of Cd, but rather the retention. It is well established that (i) the coordination environments of metal ions are a key determinant for the sense and magnitude of any isotope fractionation that is imparted by metal complexation^43^ and that (ii) cysteine-rich metallothionein binds metals via the thiol groups of this amino acid^44^. The identical δ^114/110^Cd values of the yeast transformed with *TcHMA2* and *PtMt2b* thus suggests that the former protein may also utilise thiol groups present in its structure to bind Cd. If correct, this would indicate that Cd complexation by thiol groups may be associated with little or no Cd isotope fractionation. This conclusion stands in contrast to previous investigations, which suggested that Cd–thiol complexes preferentially bind isotopically light Cd^40^.

In summary, the data strongly suggest that, to a first order, the expression of specific transporter proteins are responsible for the variations observed in Cd uptake and accumulation in different cacao clones, which impart a distinct isotopic signature. Further investigations are desirable, however, to determine (i) the binding environments for Cd in NRAMP- and HMA-family proteins and their distinct isotope fingerprints, and (ii) whether additional transporters are involved in the sequestration of Cd in cacao organs.

## Materials and methods

### Samples and initial processing

#### Cacao plants

Seeds of 19 different cacao clones (Supplementary Table S3) were obtained from International Cocoa Quarantine Centre (Reading, UK). Clones were chosen to represent a diverse range of genotypes as assessed by using a maximum-likelihood tree created from single-nucleotide polymorphism data (Supplementary Fig. S3). Seed coats were removed before planting in seed compost for germination. Two-week-old seedlings were then transferred into 4-L plastic containers containing 3 L of half-strength Hoagland solution (pH 5.2). Each container contained four seedlings. The nutrient solution was aerated for 15 min, every 2 h, and renewed every week.

Twenty-eight days after planting, plants were subjected to 20 µmol L^−1^ Cd stress (by the addition of CdCl_2_; Sigma-Aldrich, 99.99% trace metals basis) for 14 days. Plants were cultured under controlled environment conditions (28/20 °C day/night temperature, 16 h photoperiod with 60% relative humidity). After 14 days of stress, each plant was harvested and divided into the shoot and roots.

Leaves were washed with deionised water, whereas roots were submerged in 20 mmol L^−1^ Na_2_EDTA solution for 15 min to remove apoplastically bound Cd, followed by washing with deionised water. Plant material were oven-dried at 70 °C and ground using a rotor mill (Pulverisette 14, Fritsch).

#### Cloning of plant genes and their heterologous expression in yeast

The target plant genes were amplified from complementary DNA transcribed from total RNA of the *T. cacao* clone NA702 and *Populus trichocarpa* var. Trichobel, respectively. Detailed protocol of RNA isolation, reverse transcription and gene cloning is described elsewhere^22^. Sequence data for these genes are available in the NCBI GenBank database as follows: *TcNRAMP5* (accession number H615049), *TcHMA2* (MT151685), *TcHMA3* (MT151686), *TcHMA3 SV* (MT151687) and *PtMt2b* gene^45^ (MN974475). These sequences can also be found in Supplementary Note 2.

The *Saccharomyces cerevisiae* strain DY1457 was transformed with a series of pDR195GTW yeast expression vectors each containing a single plant gene as listed above. The transformations were conducted using a yeast transformation kit (Sigma-Aldrich) following the manufacturer’s instructions and pDR195 (EV). The yeast strain was also subjected to double transformation with *TcNRAMP5* in combination with *TcHMA2*, *TcHMA3* and *PtMt2b* respectively for co-expression of the encoded proteins. An EV pDR195 (EV) was used as a control.

Transformants were selected on synthetic defined medium containing 6.7 g L^−1^ yeast nitrogen base without amino acids (Thermo Fisher Scientific), 1 g L^−1^ of amino acid supplement without uracil (Sigma-Aldrich) and 2% glucose, designated as SD-U medium. A single yeast colony was inoculated into the liquid medium used in the selection process and grown to an OD_600_ of 1.0.

To determine Cd^2+^ accumulation in yeast, 50 mL of SD-U liquid culture supplemented with 2 μmol L^−1^ CdCl_2_ (Sigma-Aldrich) was inoculated with the pre-cultured cells at an initial OD_600_ of 0.01. The cells were grown at 30 °C with shaking at 250 r.p.m. for 24 h. The cells were pelleted by centrifugation, washed with cold 20 mmol L^−1^ EDTA for 10 min, rinsed three times with deionised water and dried at 70 °C for 2 days.

#### Localisation of TcNRAMP5 in yeast cells

The complete coding sequence of *TcNRAMP5*, excluding stop codon, was cloned into the pSF-TEF1_COOH-eGFP (OG4722) SnapFast yeast expression vector, which contained an eGFP (enhanced green fluorescent protein) C-terminal tag. The full coding sequence of aquaporin *PIP2A* (a plasma membrane marker) without stop codon was amplified from pm-rkCD3-1007 vector^46^ and ligated into modified OG4722 yeast expression vector (pSF-TEF1_COOH-RFP) where the C-terminal tag eGFP was replaced with RFP (red fluorescent protein). Integrity of the expression cassettes were confirmed by restriction analysis, and sequencing of promoter/gene/fluorescent tag/terminator region. The yeast (*S. cerevisiae*) strain DY1457 was transformed with both expression cassettes using the method described above. Images of live yeast cells were acquired and analysed using NIS-Elements software on a Nikon A1R confocal microscope.

### Digestion of samples and initial trace metal concentration measurements

Leaf and root samples between 50 and 100 mg were mineralised alongside spinach leaf SRM (NIST 1570a) at the Imperial College London MAGIC Laboratories in 100 mL PFA vessels with 8 mL of 15.4 M HNO_3_ and 2 mL of 30–32% H_2_O_2_ using a Milestone Ethos EZ microwave system, fitted with a SK-10 high pressure rotor. The digestion encompassed heating of the samples to 90 °C for 15 min and to 180 °C for 30 min. The concentrations of Cd were then determined on a 10% sample aliquot using inductively coupled plasma quadrupole mass spectrometry (ICP-Q-MS) either at the Open University with an Agilent 8800 triple quadrupole instrument or at the Natural History Museum London with an Agilent 7700× instrument.

The yeast cells were mineralised alongside cabbage leaf SRM (IAEA-359) for 8 h in 5 mL of 70% nitric acid (TraceSELECT™ grade, Sigma-Aldrich) in closed glass vessels at 110 °C at the Chemical Analysis Facility, University of Reading. Following digestion, the total Cd concentrations of the samples were determined on a 10% sample aliquot by ICP-Q-MS using a Thermo Scientific iCAP instrument.

### Cadmium stable isotope measurements

Sample preparation for the Cd isotope analyses was performed in the clean room facilities of the MAGIC Laboratories using distilled mineral acids, 30–32% Romil UpA^TM^ grade H_2_O_2_ and 18.2 MΩ cm H_2_O from a Millipore system. The main aliquots of each sample were first equilibrated with a ^111^Cd–^113^Cd double spike (DS) solution to obtain a ratio of DS-derived to natural Cd (S/N) ≈ 1–3. A procedure that employs anion and extraction chromatography plus liquid–liquid extraction for clean-up was employed to prepare purified Cd fractions from the samples^47^. The Cd fractions were dissolved in 0.1 M HNO_3_ to make up run solutions with Cd concentrations between 20 and 60 ng mL^−1^.

The Cd isotope compositions were determined using either a Nu Instruments Nu Plasma HR or a Nu Plasma II MC-ICP-MS (multiple collector inductively coupled mass spectrometer). Sample introduction was performed with a Cetac Aridus II or a Nu Instruments DSN desolvation system, equipped with Micromist glass nebulisers with uptake rates of ~0.12 mL min^−1^. Sensitivity for Cd was 250–300 V/(µg mL^−1^) and 600–800 V/(µg mL^−1^) on the Nu Plasma HR and Nu Plasma II, respectively, measured using Faraday cups with 10^11^ Ω resistors. To monitor instrumental drift, sample runs were bracketed by analyses of the NIST SRM 3108 Cd isotope reference material, using solutions that featured S/N ratios and Cd concentrations that closely matched the samples. Following data acquisition, the raw measured isotope ratios were processed offline with an iterative technique to solve the DS equations. The final Cd concentrations of the plant and yeast samples were obtained with the isotope dilution technique using results from the DS data reduction^48^. Further details of the data acquisition and reduction protocols are provided in previous publications^47,48^.

In the following, the Cd isotope compositions of samples are reported relative to the NIST SRM 3108 Cd isotope standard:^49^

4 $${\delta^{114/110}}{\mathrm{Cd}} = \left[ {\left( {\frac{{\left( {{\,}^{114}{\mathrm{Cd/}}^{110}{\mathrm{Cd}}} \right)_{{\mathrm{Sample}}}}}{{\left( {{\,}^{114}{\mathrm{Cd/}}^{110}{\mathrm{Cd}}} \right)_{{\mathrm{Standard}}}}}} \right) - 1} \right] \ 1000$$

For the comparison of Cd isotope compositions, the apparent isotope fractionation between two samples or reservoirs was calculated as:

5 $$\Delta ^{114/110}{\mathrm{Cd}}_{{\mathrm{A}} - {\mathrm{B}}} = {\updelta}^{114/110}{\mathrm{Cd}}_{\mathrm{A}} - {\updelta}^{114/110}{\mathrm{Cd}}_{\mathrm{B}}$$

where A and B denote the two Cd reservoirs.

## Supplementary information

Supplementary Information

## Data Availability

All data necessary for interpretation, replication and building upon the methods can be found in the article tables and electronic Supplementary information.
