# Supplementary material for: Cadmium isotope fractionation reveals genetic variation in Cd uptake and translocation by Theobroma cacao and role of natural resistance-associated macrophage protein 5 and heavy metal ATPase-family transporters
Source: Hortic Res. 2020 May 1;7:71. doi: 10.1038/s41438-020-0292-6 (PMC7193571; doi:10.1038/s41438-020-0292-6)
Supplement: Supplementary file 1 — Supplementary Information [file 41438_2020_292_MOESM1_ESM.pdf]

Supplementary Information to:

Cadmium isotope fractionation reveals genetic variation in Cd uptake and translocation by *Theobroma cacao* and role of natural resistance-associated macrophage protein 5 and Heavy Metal ATPase-family transporters

Rebekah E. T. Moore*, r.moore13@imperial.ac.uk, Tel: 020 75 94 73 32, Department of Earth Science and Engineering, Imperial College London, London SW7 2BP, UK,

Ihsan Ullah, i.ullah@reading.ac.uk, School of Agriculture, Policy and Development, University of Reading, Reading RG6 6BZ, UK

Vinicius H. de Oliveira, vho@unicamp.br, School of Agriculture, Policy and Development, University of Reading, Reading RG6 6BZ, UK. Present address: Department of Plant Biology, Institute of Biology, University of Campinas, Campinas, Sao Paulo 13083-970, Brazil

Samantha J. Hammond, sam.hammond@open.ac.uk, School of Environment, Earth and Ecosystem Sciences, The Open University, Milton Keynes MK7 6AA, UK

Stanislav Strekopytov, stanislav.strekopytov@lgcgroup.com, Imaging and Analysis Centre, The Natural History Museum, London SW7 5BD, UK. Present address: National Measurement Laboratory, LGC, Queen’s Road, Teddington, TW11 0LY, UK

Mark Tibbett, m.tibbett@reading.ac.uk, School of Agriculture, Policy and Development, University of Reading, Reading RG6 6BZ, UK

Jim M. Dunwell, j.m.dunwell@reading.ac.uk, School of Agriculture, Policy and Development, University of Reading, Reading RG6 6BZ, UK

Mark Rehkämper, markrehk@imperial.ac.uk, Department of Earth Science and Engineering, Imperial College London, London SW7 2BP, UK

Table S1. Comparison of Cd concentrations and isotope compositions obtained for reference and quality control materials and comparison with literature data and results obtained for duplicate digests and analyses of roots and leaf samples from a single Matina 1-7 plant.

Material	Type	n	[Cd] ± sd (μg g^{-1})	[Cd] Lit. (μg g^{-1})	$\delta^{114/110}$Cd ± 2sd (‰)	$\delta^{114/110}$Cd Lit. (‰)
BAM-I012[a]	Cd solution	16	--	--	–1.32±0.06	–1.33±0.04
NIST SRM 1570a[b]	Spinach leaf	4	2.709±0.01	2.876±0.058	0.44±0.06	--
IAEA-359[c]	Cabbage leaf	1	0.123	0.125±0.005	--	--
MAGIC NA-702[d]	Cacao leaf	10	107±18	102	–0.55±0.13	–0.50±0.06
Sigma Aldrich $CdCl_2$[e]	Cd additive	7	--	--	–0.36±0.04	--
Matina 1-7 Digest a	Cacao root	1	195	--	–0.71±0.05	--
Matina 1-7 Digest b	Cacao root	1	218	--	–0.65±0.07	--
Matina 1-7 Digest a	Cacao leaf	1	3.79	--	–0.18±0.06	--
Matina 1-7 Digest b	Cacao leaf	1	2.96	--	–0.23±0.05	--

n = number of individual samples that were digested and analysed. [a] The literature data are the mean result from Abouchami *et al.*, 2013.[1] [b] Cd concentration from certificate. No published $\delta^{114/110}$Cd data are available for this sample. [c] The published Cd result is an 'Information value' from the certificate that is based on results from 57 laboratories. [d] In-house quality control material prepared from cacao leaves, whereby the plant was grown in a hydroponic solution containing added Cd; the literature results for this sample are from Barraza *et al.*, 2019.[2] [e] Source of Cd that was added to the hydroponic solutions and the yeast culture medium.

Table S2: Weights of the plant tissue samples

Cacao clones	Leaf g	Root g	Stem g	Total plant g
B 5/7 [POU]	0.554	0.092	0.246	0.892
Catie 1000	0.304	0.127	0.122	0.553
CC 41 (1)	0.463	0.101	0.231	0.795
CC 41 (2)	0.685	0.128	0.189	1.002
CL 19/10	1.361	0.159	0.289	1.809
GU 207/H	0.250	0.092	0.055	0.397
GU 263/V	0.332	0.144	0.089	0.565
IMC 27	0.301	0.132	0.147	0.580
LP 1/41 [POU]	0.635	0.101	0.259	0.995
Matina 1-7	1.387	0.269	0.499	2.155
NA 702	1.181	0.198	0.341	1.720
PNG 340	0.940	0.206	0.551	1.697
POUND 12/A [POU]	0.525	0.121	0.139	0.785
RB46	0.512	0.134	0.140	0.786
RIM 189 [MEX]	1.298	0.299	0.334	1.931
SCA 9	0.668	0.122	0.262	1.053
SPA 9 [COL]	0.535	0.127	0.218	0.880
TARS 31	0.534	0.280	0.181	0.994
TSA 654	0.616	0.138	0.153	0.907
U 70 [PER]	0.247	0.068	0.084	0.399
**Mean**	**0.666**	**0.152**	**0.226**	**1.045**
**sd**	**0.361**	**0.064**	**0.127**	**0.514**

Table S3. Name, accession number and donor organization of the cacao germplasm used in the study.

Clone Name	Accession number (ICQC, R)	Donor Collection
B 5/7 [POU]	RUQ 522	ICG, T
CATIE 1000	RUQ 844	CIRAD
CC 41	RUQ 1428	ICG, T
CL 19/10	RUQ 905	ICG, T
GU 207/H	RUQ 225	CIRAD
GU 263 /V	RUQ 1545	CIRAD
IMC 27	RUQ 515	ICG, T
LP 1/41 [POU]	RUQ 353	ICG, T
MATINA 1/7	RUQ 1333	ICG, T
NA 702	RUQ 1587	ICG, T
PNG 340	RUQ 1304	CIRAD
POUND 12/A	RUQ 1458	ICG, T
RB 46 [BRA]	RUQ 134	CATIE
RIM189 [MEX]	RUQ 310	CATIE
SCA 9	RUQ 1064	ICG, T
SPA 9 [COL]	RUQ 235	ICG, T
TARS 31	RUQ 1394	USDA-TARS
TSA 654	RUQ 1029	CEPLAC/CEPEC
U 70 [PER]	RUQ 1488	UNAS

CATIE	Centro Agronómico Tropical de Investigión y Enseñanza (Costa Rica)
CEPLAC	Comissão Executiva do Plano da Lavoura Cacaueira (Brazil)
CIRAD	Centre de cooperation international en recherché agronomique pour le développement (France)
ICG, T	International Cocoa Genebank (Trinidad)
ICQC, R	International Cocoa Quarantine Centre, Reading (UK)
UNAS	Universidad Nacional Agraria de la Selva (Peru)
USDA-TARS	United States Department of Agriculture, Tropical Crops and Germplasm Research, Mayaguez (Puerto Rico)

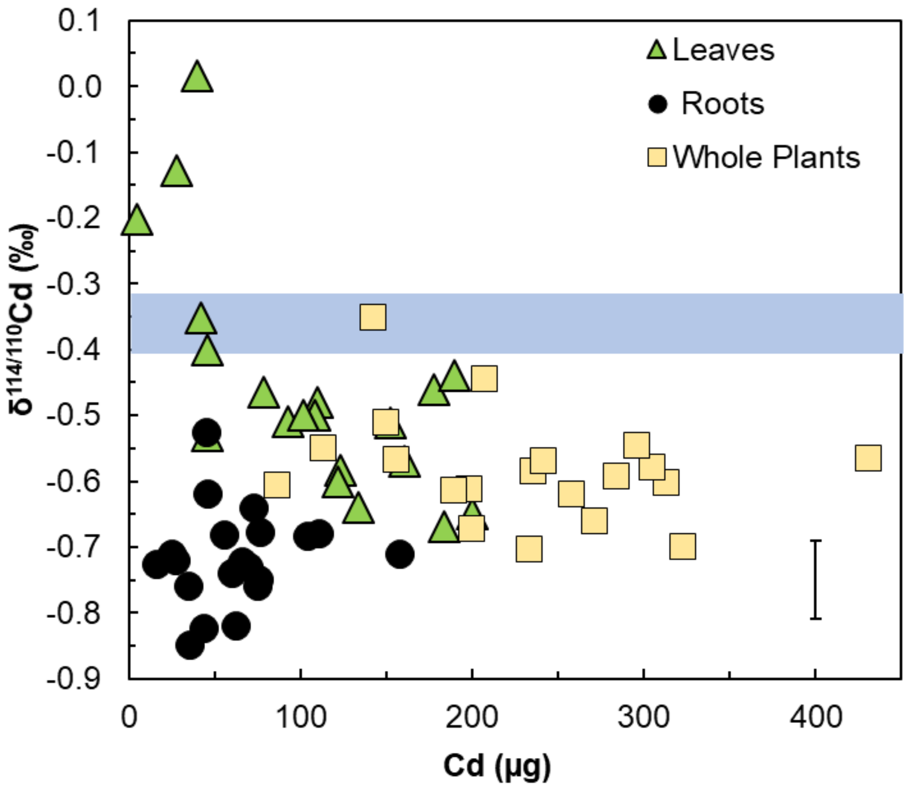

Fig. S1. Plot of $\delta^{114/110}$Cd versus Cd for the leaf, root and whole plant samples of the 19 cacao clones.

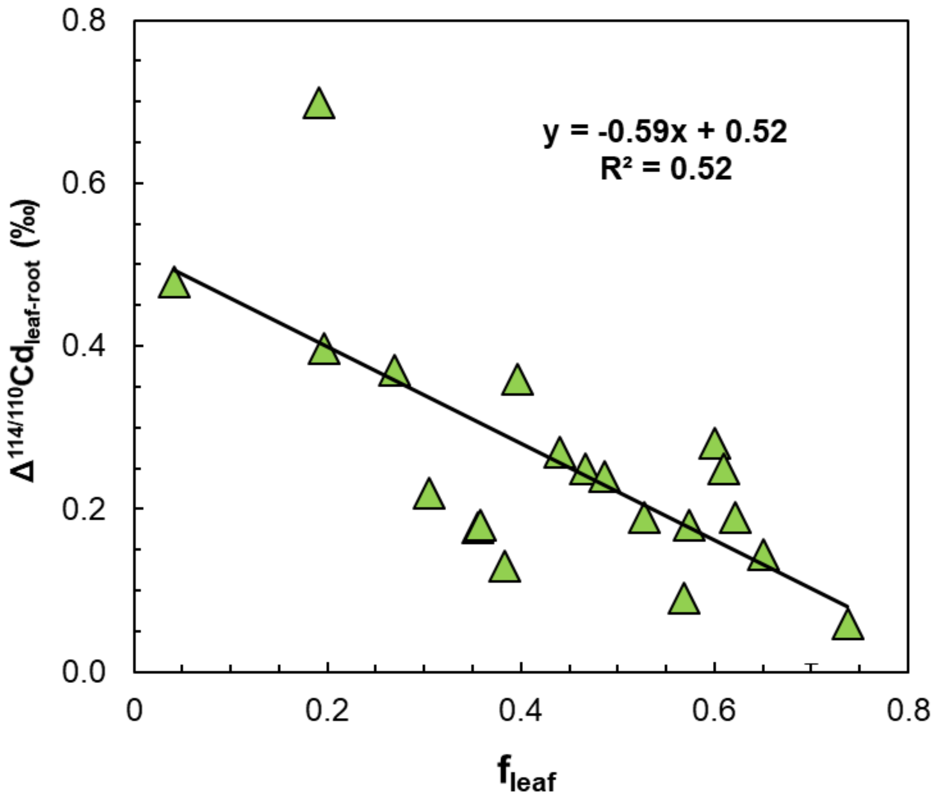

Fig. S2. Plot of $\Delta^{114/110}Cd_{leaf\text{-}root}$ versus mass fraction of Cd present in leaves (f_{leaf}) of the 19 analysed cacao clones. A scattered but clear negative correlation is apparent.

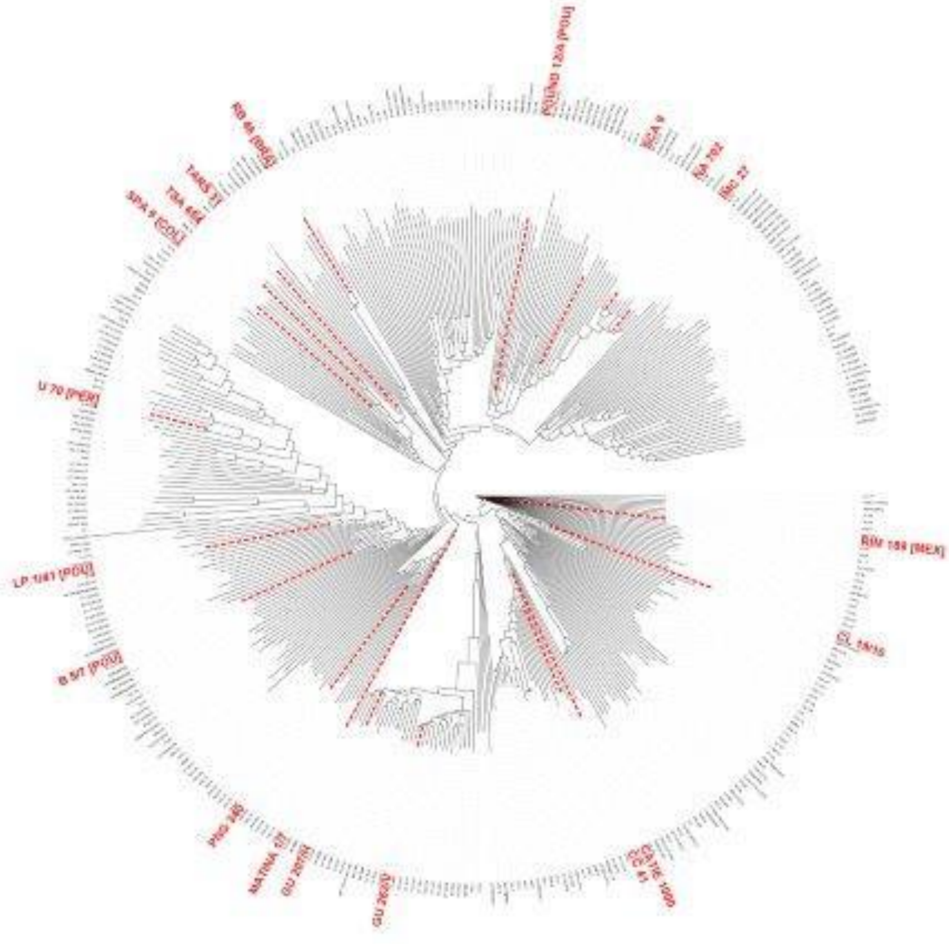

Fig S3. For germplasm selection, a maximum likelihood tree based on the JTT matrix-based model was generated in MEGA7 using the single nucleotide polymorphism (SNP) data from 380 cacao clones. The genetically diverse clones (in red) were selected using information from the evolutionary tree. The SNP data were obtained from the International Cocoa Quarantine Centre, Reading, UK.

(See http://www.icgd.reading.ac.uk/ref_search.php?refcode=ZHA14A).

Note S1: Calculation of Cd inventories for cacao stem and total plants

In this study, the stems of the cacao clones were weighed (alongside roots and leaves; Table S3) but not analysed to determine Cd isotope compositions and concentrations. To enable estimates for the Cd mass and isotope budgets of the total plants, the stems were assigned Cd concentrations $[Cd]_{stem}$ at the midpoint of the results obtained for roots and leaves:

$$[Cd]_{stem} = 0.5\ [Cd]_{root} + 0.5\ [Cd]_{leaf} \quad \text{(Eq. 1)}$$

The estimated $[Cd]_{stem}$ data were used in conjunction with $[Cd]_{root}$, $[Cd]_{leaf}$ and the weights of the plant organs (Table S3) to determine the mass fractions of Cd present in the roots, stems and leaves (f_{root}, f_{stem}, f_{leaf}). These data were then applied to determine $\delta^{114/110}Cd_{stem}$:

$$\delta^{114/110}Cd_{stem} = f_{root}\ \delta^{114/110}Cd_{root} + f_{leaf}\ \delta^{114/110}Cd_{leaf} \quad \text{(Eq. 2)}$$

This approach is firmly supported by published Cd data for plants (see below). The estimated $[Cd]_{stem}$ and $\delta^{114/110}Cd_{stem}$ values were subsequently applied to calculate the Cd concentrations, contents and isotope compositions of the total cacao plants.

To obtain uncertainty estimates for the cocoa plant Cd (isotope) mass balances, it was assumed (again in accord with literature data; see below) that deviations from the estimate provided by Eq. 1 are typically evidenced by stem compositions that are more similar to the roots than the leaves. Based on this, the most extreme stem composition that was evaluated to define the maximum ± uncertainty for each clone was thus calculated as:

$$[Cd]_{stem} = 0.75\ [Cd]_{root} + 0.25\ [Cd]_{leaf} \quad \text{(Eq. 3)}$$

whereby the respective $\delta^{114/110}Cd_{stem}$ value was subsequently determined using Eq. 2. The propagated uncertainty estimates for $\delta^{114/110}Cd_{tot}$ and $\Delta^{114/110}Cd_{leaf\text{-}tot}$ furthermore encompass the analytical errors of the Cd isotope measurements.

Use of the mass balance calculations to estimate the Cd (isotope) inventory of the cacao stems increases the uncertainties for the Cd (isotope) budgets of the total plants. Nonetheless, the approach is useful, reasonable and justified by evidence, practical constraints and the limited impact on the error propagation:

- The reduced number of Cd isotope analyses that are required with the approach significantly reduces the overall analytical cost of the project, as the measurements are associated with considerable cost and staff effort. This was helpful as the resources available for the research were limited.

- Coupled Cd isotope and concentration data that are directly comparable to the results generated in this study are available for cereals wheat, barley[3,4] and Cd accumulators (Cd tolerant *Ricinus communis*, Cd hyperaccumulator *Solanum nigrum*.[5] These literature data were evaluated carefully and used to develop and justify the approach applied here. In detail, the literature results show that the [Cd] and $\delta^{114/110}$Cd values for (i) the straw of wheat/barley are consistently intermediate between the results obtained for roots and grains; (ii) the stems of Cd accumulators are nearly always intermediate between the data for roots and leaves. On average, it is also apparent that the Cd concentrations of the stems/straw of these plants are typically roughly midway between the root and leaf/grain values, whereby most deviations reflect stem results that are somewhat closer to the root rather than the leaf/grain data. As such, the available evidence yields credence to the mass balance approach used here to estimate $[Cd]_{stem}$ and $\delta^{114/110}Cd_{stem}$ and further values derived from these parameters.
- The estimated mass fraction of Cd in the cacao clone stems varies between 15% and 48% with a mean result of f_{stem} = 28 ± 10% (1sd). As such, the stems play an important but not a dominant role in the Cd (isotope) budgets of the cacao clones. Due to this, and even using a conservative estimate for the uncertainty of $[Cd]_{stem}$ and $\delta^{114/110}Cd_{stem}$ (see above), error propagation does not generate unduly large error bars for values such as f_{leaf} and $\Delta^{114/110}Cd_{leaf\text{-}tot}$ (Fig. 3). In fact, the propagated uncertainties are sufficiently small, such that compositional and isotopic differences between the cacao clones are still clearly apparent and can be used to define systematic data trends in Figs. 2, 3 and S2.
- Finally, the validity of the mass balance approach is also demonstrated by the research findings: (i) the measured $\delta^{114/110}$Cd values show similar correlations with $[Cd]_{leaf}$ and Cd_{leaf} (which were both directly measured; Figs. 2a and S1) as well as f_{leaf} (which was calculated; Fig. 2b); (ii) a clear trend is observed for $\Delta^{114/110}Cd_{leaf\text{-}tot}$ (which combines measured and calculated data) with f_{leaf} (Fig. 5). The observed trends are unlikely to be fortuitous but most likely characterise the systematic distribution of Cd (and its isotopes) within the cacao plant, in accord with observations made in previous studies on cereals and Cd accumulators.[3–5]

Note S2: List of the gene sequences used in the study

TcHMA2_ MT151685

ATGGATGCGAATAAAAAACTGCAGAAAAGCTACTTCGATGTGCTGGGTATCTGC
TGCTCCTCGGAGGTTGCTCAGATAGAAAACATCTTAAAATCCCTTGAAGGCGTCA
AAGAAGTATCTGTGATTGTTCCTACCAGAACAGTTATTGTTCTCCATGACAATCT
CCTCGTTTCTCAGCTTCAAATAGTTAAGGCTCTAAATCAAGCAAGACTGGAAGCA
AACGTTCGAGCGCGTGGGGAGATCAAGTACCAGAAAAAATGGCCCAGCCCTTTT
GCAATAGCTTGTGGCTTGCTGCTTTTGTTTTCACTGTTGAAGTATGCATACCATCC
ATTGCAATGGCTAGCCGTAGGAGCTGTGGCCGTTGGCATCTACCCCATGCTGTTG
AAGGGCTATGCAGCCGTTAGGAATTTCAGGCTCGATATCAACATTCTTATGCTCA
GTGCAGCGATAGGGAGCATTGCAATGAAGGACTACACTGAAGCTGGCACTATCG
TCTTCCTCTTCACCACTGCGGAATGGCTTGAGTCAAGAGCAAGCCATAAGGCTAC
TGCTGTCATGTCCTCGTTGATGAGTATAGCTCCTCAGAAAGCAGTCATAGCTGAG
ACAGGAGAAGAAGTGGATGCCGATGAAGTCAAATTGAGCACTGTTCTCGCCGTT
AAGGCTGGTGAAGTTATACCCATTGATGGAATTGTTGTAGATGGGAAATGTGAA
GTGGATGAGAAAACCCTTACTGGTGAATCACTTCCAGTTACCAAAGAAAAGGAT
TCAACTGTCTGGGCTGGCACCATCAATCTAAACGGTTACATTAGTGTAAAAACTA
CTGCTGTAGCTGAAGACTGTGTAGTGGCTAAAATGGCGAAGCTAGTGGAGGAAG
CTCAAAACAACAAATCTAGAACCCAAAGATTCATAGACAAATGTGCCCAGTTCT
ACACCCCAGCAATTGTAATTGTATCAGCCGCCATTGCTGTGATTCCAGCTGCACT
GAGAGTGCATAACCTTCACAACTGGTTTCACTTAGCATTGGTTGTCTTGGTGAGT
GCATGTCCATGTGCTCTTATCCTTTCTACACCTGTTGCAAGTTTTTGTGCACTTAC
AAAAGCTGCAACATCAGGACTTCTAGTTAAGGGTGGCGACTATCTTGAAATTCTT
TCCAAAATTAAGATCACAGCTTTTGACAAAACTGGTACACTTACGAGGGGTGAA
TTTGTTGTGACCGATTTTCGATCTCTTTGTGAAGATATAAGCTTGAACACATTGCT
TTACTGGGTTTCAAGCGTTGAGAGTAAGTCCAGTCATCCAATGGCAGCTGCACTT
GTAGAATATGGAAGGTCACATTCCATCGAACCAAACCCTGAAACTGTTGAGGAT
TATCACAATTTCCCTGGGGAAGGTATTTATGGGAGGATTGATGGCAGAGATATTT
ACATTGGAAGCAGAAAAATTTCACTGAGAGCTCATGGAACAGTTCCAAGTCTAG
AAGGCAATATGATTGAAGGAAAGACCATTGGATATGTGTTTTCTGGAGCAACCC
CTGCTGGAATTTTCCGTCTTTCAGATGCTTGTCGAACTGGGGCTGCAGAGGCAGT
CAATGAGCTAAAGTCAATGGGGATAAAAGCTGCTATGCTTACAGGAGATAATCA
AGCAGCAGCCATACATGTACAAGAACAGCTAGGGAATCGTCTAGATGAAGTCCA
TGCAGAACTTCTTCCTGAAGACAAGGCAAGAATAATCGAAGAGCTTAGGAAAGA
GGGGCCAACAGCAATGATTGGAGATGGCATCAATGATGCACCTGCACTTGCTAC
AGCTGATATTGGTATTTCAATGGGCATTTCGGGTTCAGCACTTGCGACAGAGACA
GGGCATGTGATTCTCATGTCAAATGACATTAGAAAGATACCAAAAGCTATCCAA
CTGGCAAGAAAGGCACATAGGAAAGTCATTGAGAATGTCATTTTGTCAATCAGT
ACTAAGGCTGCTATTCTTGCTTTGGCCTTTGCTGGTCATCCACTTGTGTGGGCTGC
GGTTCTTGCTGATGTTGGCACATGCTTGTTGGTGATCTGTAATAGCATGCTACTTT
TGCGAGGAACACACAAACATGCAGGAAAATGTAGCAAATCTTCGGCTGCATCAC

ATACAGACAAAAAAGGATGCAAAACCAGTCATTGCCGCTTATCTGATAACCATG
AACATGCTAGCACAGACAAAAAAGTTCAAAAGCTGTGTGAGCCTAAAAGATGTT
TGTCGCAGAGGTGTGCCTCAAAATGTCAATCTAGCCCTTTCAATTCAGATTCATG
TTCAAATTCATGTGGGAGCAATAAATGTGCGGACTCAGCAAGGACACACGATGG
TTCTGTTAGTGATGGATCCCTTGAAGCAAAGCACTGTGACCAGGGTAGCTGTTGC
ATGGTCAATGACAAACGTGCAGGAAAATGTTGCAGATCTTCAACTGCATCACAT
ACAGACAAACATGGATGCAAAACCTTTCATGGCCACTCATCTCATAACCATCAAC
ATGCTATCATAGACCAAAAAGTCCAAAAGCCGTGTGCGCCTAAAAAATGTTCCT
CGCAGAAGTGTGCCGCAAAATGTCAATCTAGCCCTTTCGGTACAGATTCATGTTC
TGTGGACTCAGCAAGGGCACACGATGGTTCTGTTAATGATAGATCCCATGAAGA
AAAGCACTGTGACCAGGGAAGCTGTTGCATGGTCAATGACAAGACTGAGGCACA
CAATTTGTCCAGCAACTGTTGTTCAGGCAATCGGAGTTTGGGCTTGAACACTGAA
GATAAATGCAGAAAAGCTAGTTACTGCGTAGAAGACCAGCGAGAGACAAAAATT
GGTCATTGTCATTCTGTTCATTGCGGTGAAAACCATGTTAAGAACCACACTAACG
ATAAAGCTTTAGGAAACTTGGTAGAACATAGTTCTTCGGAGAGCCTAAATCCAA
AGGCTTATTCTCATCCACACAAGTGTTGTATAGACTACAGTGATCAGCCACCTCA
CACTGCAATAGACATTCCGATGAGTTCAGACTTTGAGGCTGCTAAAGCTCGTACG
ACTCTGGAGAAGAGAGAATTTGGTGGTTGTTGCAAGAGCTATATGAGAGAATGC
TGTGGTAAGCATGGACATTTTGGACCTGGCCTTGGAGGAGGCTTAGCAGAAATA
ACCACTGAATAG

TcHMA3_ MT151686

ATGGATGCGAATAAAAAACTGCAGAAAAGCTACTTTGATGTGCTGGGTATCTGCT
GCTCCTCGGAGGTTGCTCAGATAGAAAACATCTTAAAATCCCTTGAAGGCGTCAA
AGAAGTATCTGTGATTGTTCCTACCAGAACAGTTATTGTTCTCCATGACAATCTC
CTCGTTTCTCAGCTTCAAATAGTCAAGGCTCTAAATCAAGCAAGACTGGAAGCAA
ACGTTCGAGCGCGTGGGGAGATCAAGTACCAGAAAAAATGGCCCAGCCCTTTTG
CAATAGCTTGTGGCTTGCTGCTTTTGTTTTCACTGTTGAAGTATGCCTACCATCCA
TTGCAATGGCTAGCCGTAGGAGCTGTGGCCGTTGGCATCTACCCCATGCTTTTGA
AGGGCTATGCGGCCGTTAGGAATTTCAGGCTCGATATCAACATTCTTATGCTCAG
TGCAGTGATAGGGAGCGTTGCAATGAAGGACTACACTGAAGCTGGCACTATCGT
CTTCCTCTTCACCACTGCAGAATGGCTTGAGTCAAGAGCAAGCCATAAGGCTACT
GCTGTTATGTCCTCGTTGATGAGTATAGCTCCTCAGAAAGCAGTCATAGCTGAGA
CAGGAGAAGAAGTGGATGCCGATGAAGTCAAATTGAGCACTGTTCTCGCAGTTA
AGGCTGGTGAAGTTATACCCATTGATGGAATTGTTGTAGATGGGAAATGTGAAGT
GGATGAGAAAACCCTTACTGGTGAATCATTTCCAGTTACCAAAGAAAAGGATTC
AACTGTCTGGGCTGGCACCATCAATCTAAACGGTTACATTAGTGTAAAAACTACT
GCTGTAGCTGAAGACTGTGTAGTGGCTAAAATGGCGAAGCTAGTGGAGGAAGCT
CAAAACAGCAAATCTAAAACCCAAAGATTCATAGACAAATGTGCCCAGTTCTAC
ACCCCAGCAATTGTAATTGCATCAGCTGCCATTGCTGTGATTCCAGCTGCACTGA
GAGTGCATAACCTTCACAACTGGTTTTACTTAGCATTGGTTGTCTTGGTGAGTGC
ATGTCCATGTGCGCTTATCCTTTCTACACCTGTTGCAAGTTTTTGTGCACTTACAA

AAGCTGCAACATCAGGACTTCTAGTTAAGGGTGGCGACTATCTTGAAATTCTTTC
CAAAATTAAGATCACAGCTTTTGACAAAACTGGTACACTTACGAGGGGTGAATTT
GTTGTGACCGATTTTCGATCTCTTTGTGAAGATATAAGCTTGAACACATTGCTTTA
CTGGGTTTCAAGCGTTGAGAGTAAGTCAAGTCATCCAATGGCAGCTGCACTTGTA
GAATATGGAAGGTCACATTCCATTGAACCAAACCCTGAAACTGTTGAGGATTATC
ACAATTTCCCTGGGGAAGGTATTTATGGGAGGATTGATGGCAGAGATATTTACAT
TGGAAGCAGAAAAATATCACTGAGAGCTCATGGAACAGTTCCAAGTCTAGAAGG
CAATATGATTGAAGGAAAGACCATTGGATATGTGTTTTCTGGAGCAACCCCTGCT
GGAATTTTCAGCCTTTCAGATGCTTGTCGAACTGGGGCTGCAGAGGCAGTCAATG
AGCTAAAGTCAATGGGGATAAAAGCTGCTATGCTTACAGGAGATAATCAAGCAG
CAGCCATACATGTACAAGAACAGCTAGGGAATCGTCTAGATGAAGTCCATGCAG
AACTTCTTCCTGAAGACAAGGCAAGAATAATCGAAGAGCTTAGGAAAGAGGGGC
CAACAGCAATGATTGGAGATGGCATCAATGATGCACCTGCACTTGCTACAGCTG
ATATTGGTATTTCAATGGGCATTTCGGGTTCAGCACTTGCGACAGAGACAGGGCA
TGTGATTCTCATGTCAAATGACATTAGAAAGATACCAAAAGCTATCCAACTGGCA
AGAAAGGCACATAGGAAAGTCATTGAGAATGTCATTTTGTCAATCAGTACTAAG
GCTGCTATTCTTGCTTTGGCCTTCGCTGGTCATCCACTTGTGTGGGCTGCGGTTCT
TGCTGATGTTGGCACATGCTTGTTGGTGATCTGTAATAGCATGCTACTTTTGCGAG
GAACAGACAAACATGCAGGAAAATGTAGCAAATCTTTGGCTGCATCACATACAG
ACAAACAAGGATGCAAAACCAGTCATTGCCGCTTATCTGATAACCATGAACATG
CTAGCACAGACAAAAAAGTTCAAAAGCTGTGTGAGCCTAAAAAATGTTCGTCGC
AGAGGTGTGCCTCAAAATGTCAATCTAGCCCTTTCAATTCAGATTCATGTTCAAA
TTCATGTGGGAGCAATAAATGTGCGGACTCAGCAAGGACACACGATGGTTCTGT
CAGTGATGGATCCCTTGAAGCAAAGCACTGTGACCAGGGTAGCTGTTACATGGT
CAATGACAAACGTGCAGGAAAATGTTGCAGATCTTCAACTGCATCACATACAGA
CAAACATGGATGCAAAACCTTTCACGGCCACTCATCTCATAACCATCAACATGCT
AGCATAGACCAAAAAGTCCAAAAGCCGTGTGCGCCTAAAAAATGTTCCTCGCAG
AAGTGTGCCGCAAAATGTCAATCTAGCCCTTTCGGTACAGATTCATGTTCTGCGG
ACTCAGCAAGGGCACACAATGGTTCTGTTAGTGATAGATCCCATGAAGAAAAGC
ACTGTGACCAGGGAAGCTGTTGCATGGTCAATGACAAGACTGAGGCACACAATT
TGTCCAGCAACTGTTGTTCGGGCAATCGGAGTTTGGGCTTGAACACAGAAGATA
AATGCAGAAAAGCTAGTTACTGCGTAGAAGACCAGCGAGAGACAAAAATTGGTC
ATTGTCATTCTGTTCATTGCGGTGAAAACCATGTTAAGAACCACACTAACGATAA
AGCTTTAGGAAACTTGGTAGAACATAGTTCTTCGGAGAGCCTAAATCCAAAGGC
TTATTCTCATCCACACAAGTGTTGTATAGACTACAGTGATCAGCCACCTCACACT
GCAATAGACATTCCGATGAGTCCAAACTTTGAGGCTGCTAAAGCTCGTACGACTC
TGGAGAAGAGAGAATTTGGTGGTTGTTGCAAGAGCTATATGAGAGAATGCTGTG
GTAAGCATGGACATTTTGGACCTGGCCTTGGAGGAGGCTTAGCAGAAATAACCA
CTGAATAG

TcHMA3_SV_ MT151687

ATGGATGCGAATAAAAAACTGCAGAAAAGCTACTTTGATGTGCTGGGTATCTGCT
GCTCCTCGGAGGTTGCTCAGATAGAAAACATCTTAAAATCCCTTGAAGGCGTCAA
AGAAGTATCTGTGATTGTTCCTACCAGAACAGTTATTGTTCTCCATGACAATCTC
CTCGTTTCTCAGCTTCAAATAGTCAAGGCTCTAAATCAAGCAAGACTGGAAGCAA
ACGTTCGAGCGCGTGGGGAGATCAAGTACCAGAAAAAATGGCCCAGCCCTTTTG
CAATAGCTTGTGGCTTGCTGCTTTTGTTTTCACTGTTGAAGTATGCCTACCATCCA
TTGCAATGGCTAGCCGTAGGAGCTGTGGCCGTTGGCATCTACCCCATGCTTTTGA
AGGGCTATGCGGCCGTTAGGAATTTCAGGCTCGATATCAACATTCTTATGCTCAG
TGCAGTGATAGGGAGCGTTGCAATGAAGGACTACACTGAAGCTGGCACTATCGT
CTTCCTCTTCACCACTGCAGAATGGCTTGAGTCAAGAGCAAGCCATAAGGCTACT
GCTGTTATGTCCTCGTTGATGAGTATAGCTCCTCAGAAAGCAGTCATAGCTGAGA
CAGGAGAAGAAGTGGATGCCGATGAAGTCAAATTGAGCACTGTTCTCGCAGTTA
AGGCTGGTGAAGTTATACCCATTGATGGAATTGTTGTAGATGGGAAATGTGAAGT
GGATGAGAAAACCCTTACTGGTGAATCATTTCCAGTTACCAAAGAAAAGGATTC
AACTGTCTGGGCTGGCACCATCAATCTAAACGGTTACATTAGTGTAAAAACTACT
GCTGTAGCTGAAGACTGTGTAGTGGCTAAAATGGCGAAGCTAGTGGAGGAAGCT
CAAAACAGCAAATCTAAAACCCAAAGATTCATAGACAAATGTGCCCAGTTCTAC
ACCCCAGGGTTTCAAGCGTTGAGAGTAAGTCAAGTCATCCAATGGCAGCTGCAC
TTGTAGAATATGGAAGGTCACATTCCATTGAACCAAACCCTGAAACTGTTGAGG
ATTATCACAATTTCCCTGGGGAAGGTATTTATGGGAGGATTGATGGCAGAGATAT
TTACATTGGAAGCAGAAAAATATCACTGAGAGCTCATGGAACAGTTCCAAGTCT
AGAAGGCAATATGATTGAAGGAAAGACCATTGGATATGTGTTTTCTGGAGCAAC
CCCTGCTGGAATTTTCAGCCTTTCAGATGCTTGTCGAACTGGGGCTGCAGAGGCA
GTCAATGAGCTAAAGTCAATGGGGATAAAAGCTGCTATGCTTACAGGAGATAAT
CAAGCAGCAGCCATACATGTACAAGAACAGCTAGGGAATCGTCTAGATGAAGTC
CATGCAGAACTTCTTCCTGAAGACAAGGCAAGAATAATCGAAGAGCTTAGGAAA
GAGGGGCCAACAGCAATGATTGGAGATGGCATCAATGATGCACCTGCACTTGCT
ACAGCTGATATTGGTATTTCAATGGGCATTTCGGGTTCAGCACTTGCGACAGAGA
CAGGGCATGTGATTCTCATGTCAAATGACATTAGAAAGATACCAAAAGCTATCC
AACTGGCAAGAAAGGCACATAGGAAAGTCATTGAGAATGTCATTTTGTCAATCA
GTACTAAGGCTGCTATTCTTGCTTTGGCCTTCGCTGGTCATCCACTTGTGTGGGCT
GCGGTTCTTGCTGATGTTGGCACATGCTTGTTGGTGATCTGTAATAGCATGCTACT
TTTGCGAGGAACAGACAAACATGCAGGAAAATGTAGCAAATCTTTGGCTGCATC
ACATACAGACAAACAAGGATGCAAAACCAGTCATTGCCGCTTATCTGATAACCA
TGAACATGCTAGCACAGACAAAAAAGTTCAAAAGCTGTGTGAGCCTAAAAAATG
TTCGTCGCAGAGGTGTGCCTCAAAATGTCAATCTAGCCCTTTCAATTCAGATTCA
TGTTCAAATTCATGTGGGAGCAATAAATGTGCGGACTCAGCAAGGACACACGAT
GGTTCTGTCAGTGATGGATCCCTTGAAGCAAAGCACTGTGACCAGGGTAGCTGTT
ACATGGTCAATGACAAACGTGCAGGAAAATGTTGCAGATCTTCAACTGCATCAC
ATACAGACAAACATGGATGCAAAACCTTTCACGGCCACTCATCTCATAACCATCA
ACATGCTAGCATAGACCAAAAAGTCCAAAAGCCGTGTGCGCCTAAAAAATGTTC
CTCGCAGAAGTGTGCCGCAAAATGTCAATCTAGCCCTTTCGGTACAGATTCATGT

TCTGCGGACTCAGCAAGGGCACACAATGGTTCTGTTAGTGATAGATCCCATGAA
GAAAAGCACTGTGACCAGGGAAGCTGTTGCATGGTCAATGACAAGACTGAGGCA
CACAATTTGTCCAGCAACTGTTGTTCGGGCAATCGGAGTTTGGGCTTGAACACAG
AAGATAAATGCAGAAAAGCTAGTTACTGCGTAGAAGACCAGCGAGAGACAAAA
ATTGGTCATTGTCATTCTGTTCATTGCGGTGAAAACCATGTTAAGAACCACACTA
ACGATAAAGCTTTAGGAAACTTGGTAGAACATAGTTCTTCGGAGAGCCTAAATC
CAAAGGCTTATTCTCATCCACACAAGTGTTGTATAGACTACAGTGATCAGCCACC
TCACACTGCAATAGACATTCCGATGAGTCCAAACTTTGAGGCTGCTAAAGCTCGT
ACGACTCTGGAGAAGAGAGAATTTGGTGGTTGTTGCAAGAGCTATATGAGAGAA
TGCTGTGGTAAGCATGGACATTTTGGACCTGGCCTTGGAGGAGGCTTAGCAGAA
ATAACCACTGAATAG

TcNRAMP5_MH615049

ATGGGAAGTTTGCAGCAGCAAGCGACTGACCTTGCACTTCCAAAATCATGGGGT
GGAGGGAGCAACTGTATAGCTGCTGTCAATGTGGAGGGAAGCACCCCGGAATCC
TTCCCTAGTAACGACAACAAAAGCAGTGATCATGATCATGACCCAGATCATGAG
AAACCTGGATGGAGAAAGTTTTTGTCATTTGTAGGCCCTGGTTTCCTTGTCTCATT
AGCTTATCTTGATCCTGGCAACCTGGAAACTGATATGCAAGCAGGAGCCAACCA
TGGATATGAGCTACTGTGGGTGGTGTTAATTGGATTAGTGTTTGCTCTCATAATCC
AGTCTCTTGCTGCTAATCTTGGAGTCAGCACTGGCAAGCACTTATCAGAATTGTG
CAAGGCTGAGTACCCAGTATTGATCAAATATTGCCTGTGGTTGCTTGCAGAGCTT
GCTGTCATAGCTGCTGATATTCCTGAAGTGATTGGGACAGCCTTTGCATTAAATA
TACTGTTTCATGTCCCAGTTTGGGCTGGGGTTCTCTGCACTGGTTTAAGCACTCTC
CTCCTCCTGAGCCTGCAAAGATATGGGATAAGGAAGCTGGAAATGCTGATAGCG
GTGATGGTTTTTGTAATGGCCGCATGTTTCTTTGGGGAAATGAGTTATGTAAAAC
CTCCGGCGACTGGTGTTCTTAAGGGCATGTTTGTGCCCAAGCTTTCCGGCCAAGG
AGCCACCGGCGACGCTATTGCCCTCTTGGGCGCCCTTGTTATGCCGCACAATCTC
TTTCTCCACTCTGCTCTCGTGCTCTCCAGAAAAGTACCCAACTCTGTTCGTGGCAT
CAATGCTGCGTGTAGATATTTCCTGATGGAGAGCGGCTTCGCCTTGTTTGTGGCG
TTTTTGATCAACGTAGCGGTTGTCTCGGTGACGGGCACCGTCTGCTTAGCCGATA
ATCTCTCTAGTGAAGACCAGGATCGTTGCAGCAATCTTACCCTCGACTCTGCTTC
TTTCATGCTCCAGCATGTGCTGGGAAAGTCTAGCTCAACCCTTTACGCCGTTGCA
CTTCTTGCCTCGGGGCAAAGCTCTACAATTACAGGCACTTATGCAGGACAATTTA
TCATGCAGGGCTTCTTGGATCTTAAAATGAAGAAATGGGTGAGGAACTTAATGA
CTAGGAGCATTGCCATTACACCCAGTCTTATTGTGTCCATCATTGGTGGATCTCA
AGGGGCCGGTAGACTAATCATCATTGCATCGATGATTCTATCATTTGAGCTGCCA
TTTGCTCTCATCCCACTTCTGAAATTCAGTAGCAGCTCAACCAAGATGGGACCGT
ACAAGAATTCTATATATATCATTGTGATTTCATGGATACTAGGCCTTGGAATCAT
CGGTATCAACGTCTATTACTTGAGCACAGCCTTTGTAGGTTGGTTGATTCACAAT
GATTTACCAAAAGTCGGAAATGTTTTCATCGGAATCATCGTGTTTCCTCTAATGG
CCATCTACATCCTGTCAGTCATCTATCTAACCTTCCGAAAGGACACTGTTGTAAC
ATATATCGAGCCCGACAAGAACGACCCAACTGCCCAAGCTCGGATGGAGAGTGG

GCTCAGCAACCTTGACGGGTCCTTCCGGGCAGACAATGTCCCTTACAGGCAGGAT
TTAGCTGACATACCACTGCCAGAGTAG

PtMt2b_ MN974475

ATGTCTTGCTGTGGAGGAAACTGTGGCTGCGGCTCTGGATGCAAGTGCGGCAGT
GGCTGCAATGGATGCAGCATGTACCCAGACTTGAGTTTCTCCGAGACCACCACA
AGTCAGACAATCATTGCTGGTGTAGCTCCAGTTAGGATGTTCTACGAGAGCTCTG
AGATGAACTTTGGTGCTGAGAATGGCTGCAAATGTGGATCAAACTGCACCTGTG
ATCCATGCTCCTGCAAATGA

References

1. Abouchami, W. *et al.* A Common Reference Material for Cadmium Isotope Studies - NIST SRM 3108. *Geostand. Geoanalytical Res.* **37**, 5–17 (2013).

2. Barraza, F. *et al.* Cadmium isotope fractionation in the soil – cacao systems of Ecuador: a pilot field study. *RSC Adv.* **9**, 34011–34022 (2019).

3. Wiggenhauser, M. *et al.* Cadmium isotope fractionation in soil–wheat systems. *Environ. Sci. Technol.* **50**, 9223–9231 (2016).

4. Imseng, M. *et al.* Towards an understanding of the Cd isotope fractionation during transfer from the soil to the cereal grain. *Environ. Pollut.* **244**, 834–844 (2019).

5. Wei, R. *et al.* Stable isotope fractionation during uptake and translocation of cadmium by tolerant *Ricinus communis* and hyperaccumulator *Solanum nigrum* as influenced by EDTA. *Environ. Pollut.* **236**, 634–644 (2018).
